# Cas12a target search and cleavage on force-stretched DNA†

**DOI:** 10.1039/d1cp03408a

**Published:** 2021-08-26

**Authors:** Marialucrezia Losito, Quentin M. Smith, Matthew D. Newton, Maria Emanuela Cuomo, David S. Rueda

**Affiliations:** Department of Infectious Disease, Section of Virology, Faculty of Medicine, Imperial College London London W12 0NN UK david.rueda@imperial.ac.uk; Single Molecule Imaging Group, MRC London Institute of Medical Sciences London W12 0NN UK; Discovery Sciences, AstraZeneca Cambridge CB4 0WG UK; Oncology R&D, AstraZeneca Cambridge CB2 0RE UK Emanuela.Cuomo@astrazeneca.com

## Abstract

Using optical tweezers, we investigate target search and cleavage by CRISPR–Cas12a on force-stretched λ-DNA. Cas12a uses fast, one-dimensional hopping to locate its target. Binding and cleavage occur rapidly and specifically at low forces (≤5 pN), with a 1.8 nm rate-limiting conformational change. Mechanical distortion slows diffusion, increases off-target binding but hinders cleavage.

## Abstract 2

CRISPR-Cas12a target search and cleavage on force-stretched *λ*-DNA using optical tweezers.

## Graphical abstract

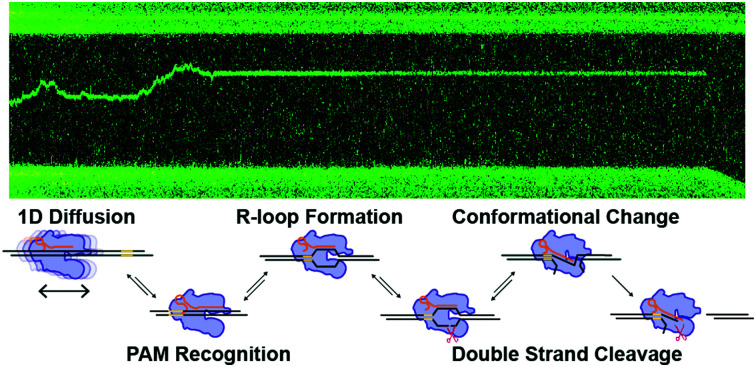

CRISPR–Cas (clustered regularly interspaced short palindromic repeats and CRISPR-associated proteins) complexes are RNA-guided endonucleases that protect bacteria against invading bacteriophages.^1,2^ Over the past decade, these complexes have gained popularity for their applications in targeted genome editing.^3^ However, the presence of spurious off-target effects has hindered some therapeutic applications.^4,5^ More recently, Cas12a^6^ (a type V CRISPR effector, Fig. 1A) has surfaced as a promising alternative over the well-known Cas9 for genomic applications^7,8^ and diagnostics^9^ because it presents fewer off-target effects in cells.^10,11^

**Fig. 1:**
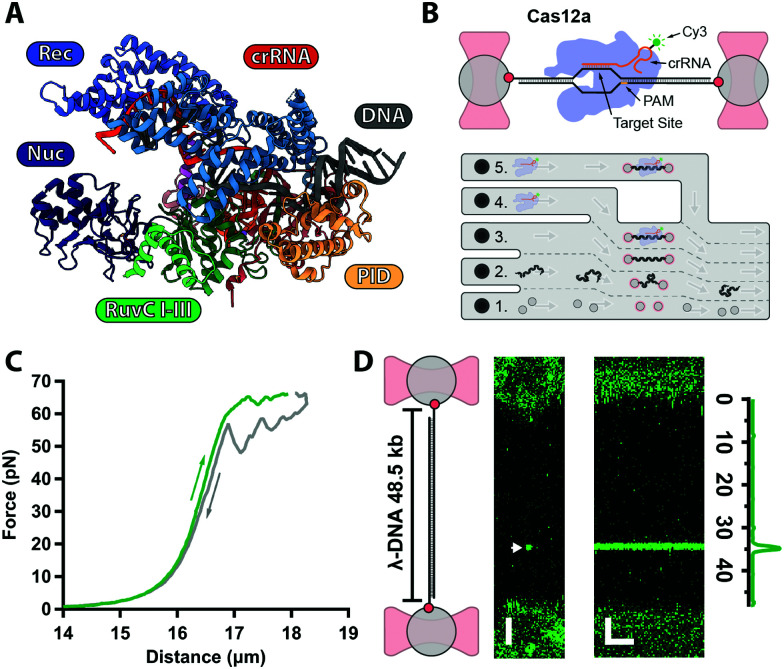
(A) Structure of AsCas12a complex with crRNA (orange) and target DNA (grey) with protein domains highlighted (PDB: 5B43). (B) Biotinylated λ-DNA tethered between optically trapped beads, with bound Cy3-labelled Cas12a complex (top). Microfluidics (bottom): (1) beads channel; (2) DNA channel; (3) buffer-only channel; (4) Cas12a in Ca^2+^-buffer; (5) Cas12a in Mg^2+^-buffer. (C) Force–distance curve of a single λ-DNA molecule stretched (green) from 0–65 pN and relaxed (grey) down to 0 pN. (D) 2D-confocal image of λ-DNA at 5 pN with a single Cy3–Cas12a complex bound at expected target site (33.5 kb, arrow) in Ca^2+^-buffer (ESI†). Kymograph shows Cas12a stably bound on target for several minutes. Genomic location analysis confirms on-target binding. Scale bars = 2 μm and 2 min.

Cas12a is a multi-domain protein (Fig. 1A) comprised of a Recognition domain (REC), a protospacer-adjacent motif (PAM, 5′-TTTV-3′, with V = A/C/G) interacting domain (PID), an inactive Nuclease domain (NUC) and an active Nuclease domain (RuvC). Unlike Cas9, Cas12a utilizes a single 43 nt-long CRISPR–RNA (crRNA), and RuvC cleaves both the non-target (NTS) and target (TS) strands sequentially, leaving a 5–8 nt overhang.^6,12^ Numerous atomic resolution structures^13–19^ have shown that the NTS interacts firsts with RuvC, whereas the TS must undergo a large conformational change to reach the RuvC active site. Despite these structures, the kinetics and target search and cleavage mechanisms of Cas12a are still not fully characterised.

Prior single-molecules studies^19–23^ have confirmed that Cas12a functions differently than Cas9. Cas12a diffuses one-dimensionally to locate targets,^21^ followed by R-loop formation,^22^ conformational activation^19^ and sequential staggered cleavage of the target.^20^

Here, we use our previously developed optical tweezers assay^24^ to further investigate the Cas12a target search and cleavage mechanism on mechanically-stretched λ-DNA. To monitor Cas12a–DNA interactions in real-time with single-molecule resolution, we assembled Cas12a complexes with a Cy3-labeled crRNA designed to target a unique sequence at 33.5 kb on λ-DNA (Fig. 1B and ESI†). Biotinylated-λ-DNA was tethered between two ∼4.9 μm polystyrene beads through biotin–streptavidin interactions and stretched under 5 pN force (Fig. 1B), to reach the DNA's contour length (∼16 μm) without altering base pairing (Fig. 1C). In the Ca^2+^-buffer channel (ESI,† Fig. S1B) to prevent cleavage, we observed a binding event at the expected location for several minutes (Fig. 1D), consistent with previous observations that Cas12a complex binds target DNA with very high affinity.^12^ No binding was observed with Cy3-crRNA alone (ESI†), confirming that such events represent stable binding of the holoenzyme complex.

Next, we tested whether Cas12a was active on force-clamped λ-DNA (3 pN) by assembling the labelled complex in the Mg^2+^-buffer channel (ESI,† Fig. S1B). Fluorescent labelling did not affect activity in control bulk assays.^20^ A single Cas12a complex bound its specific target, as expected, and cleavage was observed at 20 s as a drop in the force between the beads and a displacement of the force-clamped bead (Fig. 2A). We approximate the cleavage rate constant (*k*_cleave_ = 0.021 ± 0.001 s^−1^) by measuring the dwell time at the target site from initial binding to cleavage (*n* = 22). We repeated the measurements at forces ranging 2–7 pN (Fig. 2B). The dwell time between binding and cutting increased linearly with force, and the corresponding rate constants decreased exponentially, indicating that force hinders cleavage. At 10 pN or higher, we could not observe cleavage within our 800 s experimental window. A fit to the Bell–Evans equation^25,26^ (Fig. 2B), yields the zero-force cleavage rate constant (*k*_cleave_(0) = 0.08 ± 0.01 s^−1^), and the distance to the transition state (*x*^‡^ = 1.8 ± 0.3 nm). The zero-force cleavage rate constant is consistent with prior bulk measurements.^12,27^ The distance to the transition state indicates a large, rate limiting conformational change between binding and complete cleavage. This is consistent with the expected movement of the Rec domain bringing the TS towards the cleavage site in RuvC. Interestingly, these domains are ∼2 nm apart in the various published structures.^13–19^ This conformational change is also in agreement with prior single-molecule FRET experiments^19^ and molecular dynamics calculations.^28^

**Fig. 2:**
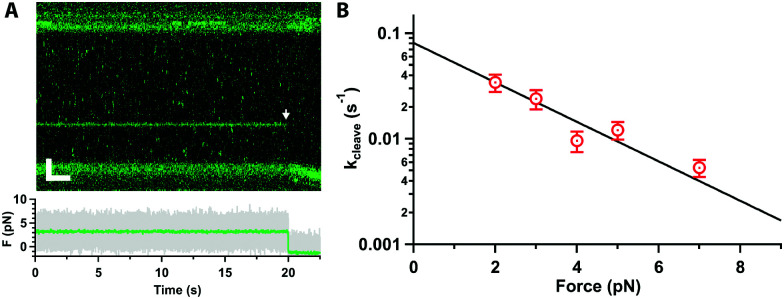
(A) Kymograph of force clamped λ-DNA (3 pN) in 10 mM Mg^2+^ and 100 mM Na^+^ with Cas12a bound on target. Cleavage is observed at 20 s as fluorescence loss (arrow), displacement of the trapped bead, and drop in the force (bottom). Scale bars = 2 μm, 2 s. (B) Cleavage rate constant as a function of force (average and standard error of the mean (s.e.m.), *n* ≥ 17 each), and fit to the Bell–Evans equation (line).

In approximately half of the traces (*n* = 26/50), the Cas12a complex first binds away from the target site, followed by random and bidirectional diffusion until the target is located (Fig. 3A, star), consistent with prior single-molecule observations.^21^ The complex can pass the target site multiple times before binding it tightly and cleaving (Fig. 3A, arrow). Likely, Cas12a cannot bind the target tightly until the PID (Fig. 1A) recognizes the PAM sequence. To characterise the diffusional behaviour of the Cas12a complex, we extract diffusion trajectories from the kymographs (Fig. 3B) and calculate their Mean Square Displacement (MSD, Fig. 3C). The trajectories show that diffusing complexes can travel thousands of base pairs in just a few seconds (Fig. 3C), with average speeds ∼1 kbp per s compared with the static, target-bound molecules (compare red and grey trajectories, Fig. 3B-D). The slope of the initial linear rise of the MSD curves yields the diffusion coefficient,^29^*D*_100_ = (1.6 ± 0.9) × 10^6^ bp^2^ s^−1^, one order of magnitude slower than a previously reported value.^21^ A possible explanation for this discrepancy is that DNA held under force (5 pN) affects diffusion (*vide infra*). In addition, the previous study used SYTOX-orange stained DNA, a DNA intercalator that crucially affects its biomechanical properties by increasing base pair separation, malleability and decreasing its persistence length,^30^ which could affect diffusion.

**Fig. 3:**
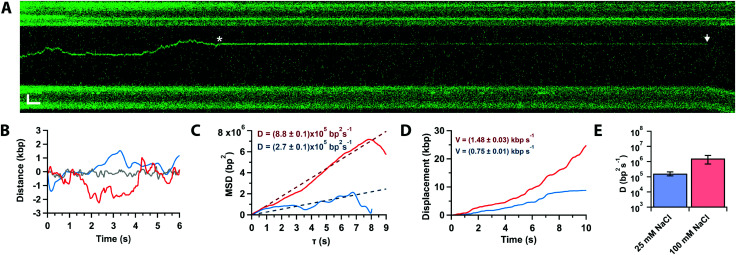
(A) Kymograph of force-clamped λ-DNA (4 pN) in 10 mM Mg^2+^ and 100 mM Na^+^ with a diffusing Cas12a complex. The complex diffuses twice past the target site (star) before binding tightly. Cleavage is observed 75 s later (arrow). Scale bars = 2 μm, 2 s. (B) Trajectories of target bound (grey) and diffusing of Cas12a complex in 10 mM Ca^2+^ and 100 mM (red) or 25 mM (blue) Na^+^. (C) Mean-square displacement analysis and diffusion coefficients of trajectories in (B) at 25 (blue) and 100 mM (red) Na^+^. (D) Cumulative displacement analysis of trajectories in (B) at 25 (blue) and 100 mM (red) Na^+^, and average diffusion velocities. (E) Diffusion coefficients in 25 and 100 mM Na^+^ (average and s.e.m., *n* = 20 each).

To inquire about the diffusion mechanism (sliding or hopping),^29^ we measured the diffusion coefficient at low ionic strength (25 mM Na^+^). The data show that the diffusion coefficient decreases by an order of magnitude with ionic strength (*D*_25_ = (1.7 ± 0.5) × 10^5^ bp^2^ s^−1^, Fig. 3D), consistent with Cas12a hopping on the DNA, also in agreement with the previous single-molecule observations.^21^

Finally, we investigated how increasing the tension on the DNA affects Cas12a binding. At low forces (Fig. 4A, ≤20 pN), we obtained primarily on-target binding with only a few off-target bound molecules that diffuse randomly on the stretched DNA (Supplementary Movie, ESI†). Increasing the force (Fig. 4A, ≥25 pN), results in additional binding events at off-target locations. To quantify this, we determined the number of off-target binding events per 10 000 bp as a function of force (Fig. 4B). The number of off-target bound complexes increases sigmoidally, which can be fit to the Hill-equation to yield a saturation of 2.9 ± 1.4 complexes bound per 10 000 bp, and a mid-point force of 37 ± 15 pN. Interestingly, we also observed that, as the force increased to 30 pN, the diffusion coefficient decreases by 30-fold (*D* = (0.5 ± 0.2) × 10^5^ bp^2^ s^−1^, Fig. 4C). Bound Cas12a complexes eventually cease to diffuse and remain bound to their initial (off-target) binding site on the DNA, comparable to the behaviour of Cas9.^24^ These data raise the interesting possibility that Cas12a “probes” the DNA sequence at every hop as it diffuses along the DNA. Since DNA stretching facilitates melting^24^ and R-loop formation,^22^ it is possible to speculate that mechanically distorting the DNA increases the dwell time at every “hop”, thereby decreasing the observed diffusion coefficient, which in turn results in frequent off-target Cas12a binding in a force-dependent fashion. Nonetheless, the force-induced cleavage inhibition observed above (Fig. 2) suggests that these off-target binding events may not result in off-target cleavage. Indeed, we never observed cleavage at these forces in Mg^2+^-buffer.

**Fig. 4:**
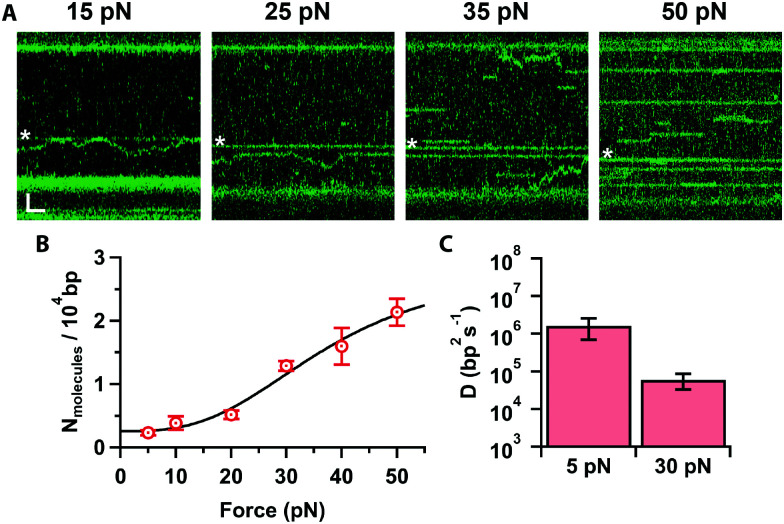
(A) Kymographs of force stretched λ-DNA (15–50 pN) in 10 mM Ca^2+^ and 100 mM Na^+^, showing Cas12a bound on target (star) and off targets. Scale bars = 2 μm, 1 s. (B) Number of molecules bound per 10 000 bp as a function of force (average and s.e.m., *n* = 7 each), and fit to the Hill equation (line). (C) Cas12a complex diffusion coefficients at 5 and 30 pN (average and s.e.m., *n* = 20 each).

## Conclusions

In recent years, Cas12a^6^ has emerged as a salient alternative for genome editing applications to Cas9 because it exhibits fewer off-target effects in cells^10,11^ and staggered cleavage.^6,12^ While Cas12a has already been investigated by some single-molecule studies,^19–22^ several mechanistic aspects remain poorly characterized. Here, we have used correlative single-molecule optical tweezers with fluorescent detection to further investigate the target search and cleavage mechanism of Cas12a on mechanically stretched λ-DNA.^24^ We find that the complex binds primarily away from the target on the DNA and diffuses randomly and bidirectionally until it engages with its target (Fig. 3A). Target recognition likely requires the PID (Fig. 1A) to recognize the PAM sequence as it diffuses over it, which can entail several attempts (Fig. 3A). Once bound at the target site, the duplex is melted, the R-loop formed, and cleavage is observed within seconds (Fig. 2A). Stretching the DNA with force hinders cleavage, which reveals a large amplitude (∼1.8 nm, Fig. 2B) rate-limiting conformational change. This conformational change could involve a “closing” of the enzyme to bring the TS in Rec (Fig. 1A) into the active site in RuvC (Fig. 1A), and is consistent with known structures^13–19^ and prior observations.^19,28^

**Fig. 5:**
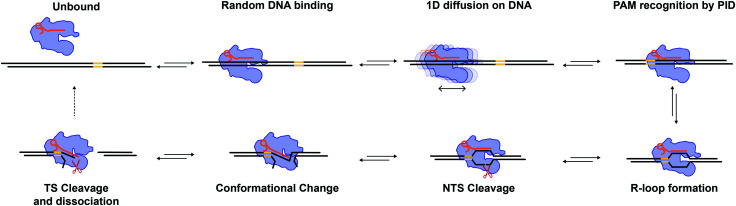
Model for Cas12a target search and cleavage. Cas12a binds DNA at a random location and undergoes 1D diffusion to locate its target. The PID recognizes the target's PAM sequence, which triggers a cascade of conformational changes to unwind the DNA duplex and form the R-loop. The NTS interacts with the RuvC domain triggering the first cleavage event. A rate limiting conformational change brings the TS into the RuvC active site enabling staggered TS cleavage. The DSB is complete and Cas12a is recycled for another round of catalysis.

Our diffusion analysis supports a search mechanism that involves hopping on the DNA, as previously proposed.^21^ The observed slow diffusion at higher forces (Fig. 4C), is consistent with the idea that Cas12a probes the DNA sequence at every hop. In this scenario, force-facilitated DNA melting, and R-loop formation would result in longer dwell-times between hops. Therefore, Cas12a likely toggles between “open” and “closed” conformations while hopping. PAM-recognition by PID likely triggers a cascade of conformational changes that result in R-loop formation and staggered cleavage of the target (Fig. 5).

Interestingly, we also observe that off-target binding increases markedly at higher forces (>20 pN), a behaviour akin of Cas9.^24^ This observation is consistent with the idea that force stretching lowers the DNA melting barrier and facilitates R-loop formation, even in the presence of multiple mismatches. However, Cas12a exhibits fewer off-target effects *in vivo*. The force-induced cleavage inhibition shown here implies that those off-target bound complexes do not cleave, which will have minimal implications for off-target activity *in vivo*. Therefore, our results shed new light into the specificity mechanism of Cas12a. The opposing effect of DNA unwinding on off-target binding and cleavage provides a mechanistic basis for Cas12a specificity. In physiological environments, negatively supercoiled DNA, which is also underwound, may experience similar off-target binding, but it is also unlikely to be cleaved. It is possible to speculate that bacteria have evolved this mechanism to distinguish between their naturally supercoiled genomes, and the linearised genome of invading phages, which would be readily cleaved. Further studies will test these hypotheses directly.

## Conflicts of interest

The authors declare no competing financial interests.

## Supplementary Material

CP-023-D1CP03408A-s001

CP-023-D1CP03408A-s002
