# Supplementary material for: Cas12a target search and cleavage on force-stretched DNA
Source: Phys Chem Chem Phys. 2021 Aug 26;23(47):26640–4. doi: 10.1039/d1cp03408a (PMC8653695; doi:10.1039/d1cp03408a)
Supplement: CP-023-D1CP03408A-s001 [file CP-023-D1CP03408A-s001.pdf]

Electronic Supplementary Material (ESI) for Physical Chemistry Chemical Physics.
This journal is © the Owner Societies 2021

Electronic Supplementary Information

for

Cas12a Target Search and Cleavage on Force-Stretched DNA

Marialucrezia Losito,†[a,b,c] Quentin M. Smith,†[a,b], Matthew D. Newton[a,b], M. Emanuela Cuomo*[,d] and David S. Rueda*[a,b]

[a] *Department of Infectious Disease, Section of Virology, Faculty of Medicine, Imperial College London, London W12 0NN, UK.*

[b] *Single Molecule Imaging Group, MRC London Institute of Medical Sciences, London W12 0NN, UK.*

[c] *Discovery Sciences, AstraZeneca, Cambridge CB4 0WG, UK.*

[d] *Oncology R&D, AstraZeneca, Cambridge CB2 0RE, UK.*

† Equally contributingfinal authors.

* Address correspondence to: david.rueda@imperial.ac.uk or Emanuela.Cuomo@astrazeneca.com

Methods

RNA purification and labeling. crRNA guide was synthesized by IDT as a 43nt RNA oligo with an internal Amino-Modifier-C6-dT (Supplementary Table 1). Oligo was labeled with Cy3-NHS ester and HPLC purified as described[1]. RNA oligo was stored at 5μM stock in water (-20°C).

Protein storage and complexing. Cas12a (IDT) and was stored at -80°C and 5 μM in storage buffer (20mM Tris-HCl, pH 7.5, 150mM KCl, 10% glycerol and 1 mM tris(2-carboxyethyl)phosphine). Working fractions were aliquoted, snap frozen in liquid nitrogen.

Optical tweezers experiments. Optical tweezer experiments were performed on a C-trap (LUMICKS A.G., Amsterdam) with integrated confocal microscopy and microfluidics, as described[2]. Channels in the microfluidics chip were first passivated with BSA (0.1% w/v in PBS) and Pluronics F128 (0.5% w/v in PBS), both flowed through over ≥30min. Bacteriophage λ-DNA was labeled at either end with biotin as described[2] and attached to 4.89 μm SPHERO Streptavidin Coated polystyrene particles at 0.005% w/v using the laminar flow cell. DNA integrity was verified before each experiment by generation of force-extension curves from 0-60 pN (Fig. 1C). For confocal imaging, 532 nm laser was used for Cy3 excitation, with emission detected in three channels with blue (512(25) nm), green (585(75)) nm and red (640LP) filters. Experiments were performed at an equilibrium temperature of 30˚C measured at the objectives.

Cas12a complex was assembled by first heat-annealing the Cy3-crRNA (5 μM) at 95°C for 2min, followed by slow cooling (~10 min) at room temperature. Then, Cy3-crRNA (250 nM) was complexed with Cas12a (either 250nM or 1μM) at room temperature for 30min in complexing buffer (50 mM Tris–HCl, pH 7.5, 100 mM or 25 mM NaCl, 10 mM $CaCl_2$ or $MgCl_2$, 0.2 mg.ml^{-1} BSA, 0.5 mM DTT), and subsequently diluted to 250 pM with either Ca^{2+}-buffer (50 mM Tris–HCl, pH 7.5, 100 mM or 25 mM NaCl, 10 mM $CaCl_2$, 0.2 mg ml^{-1} BSA, 0.5 mM DTT, 5 mM protocatechuic acid (PCA), 100 nM protocatechuic dioxygenase (PCD) and saturated Trolox) or Mg^{2+}-buffer (50 mM Tris–HCl, pH 7.5, 100 mM or 25 mM NaCl, 10mM $MgCl_2$, 0.2 mg ml^{-1} BSA, 0.5 mM DTT, 5 mM PCA, 100 nM PCD and saturated Trolox).

Diffusion and cleavage analysis. Kymographic projections were exported from Bluelake (LUMICKS A.G., Amsterdam) in .h5 format. For diffusion analysis, we used a combination of custom python (https://github.com/singlemoleculegroup) and Igor Pro 8 (Wavemetrics) scripts. Cas12a diffusion events were tracked and quantified using the python single particle tracking algorithm, and resulting trajectories were further processed in Igor Pro 8 to determine velocity trajectories and mean squared displacement plots.

Supplementary Table 1

**Oligo Name**	**Sequence** (5' -> 3')
Cpf1_crRNA_33.5	AAUUUCUACU CU/AmC6T/GUAGAUA TTCAGATGAT ATGACTATCA AGG

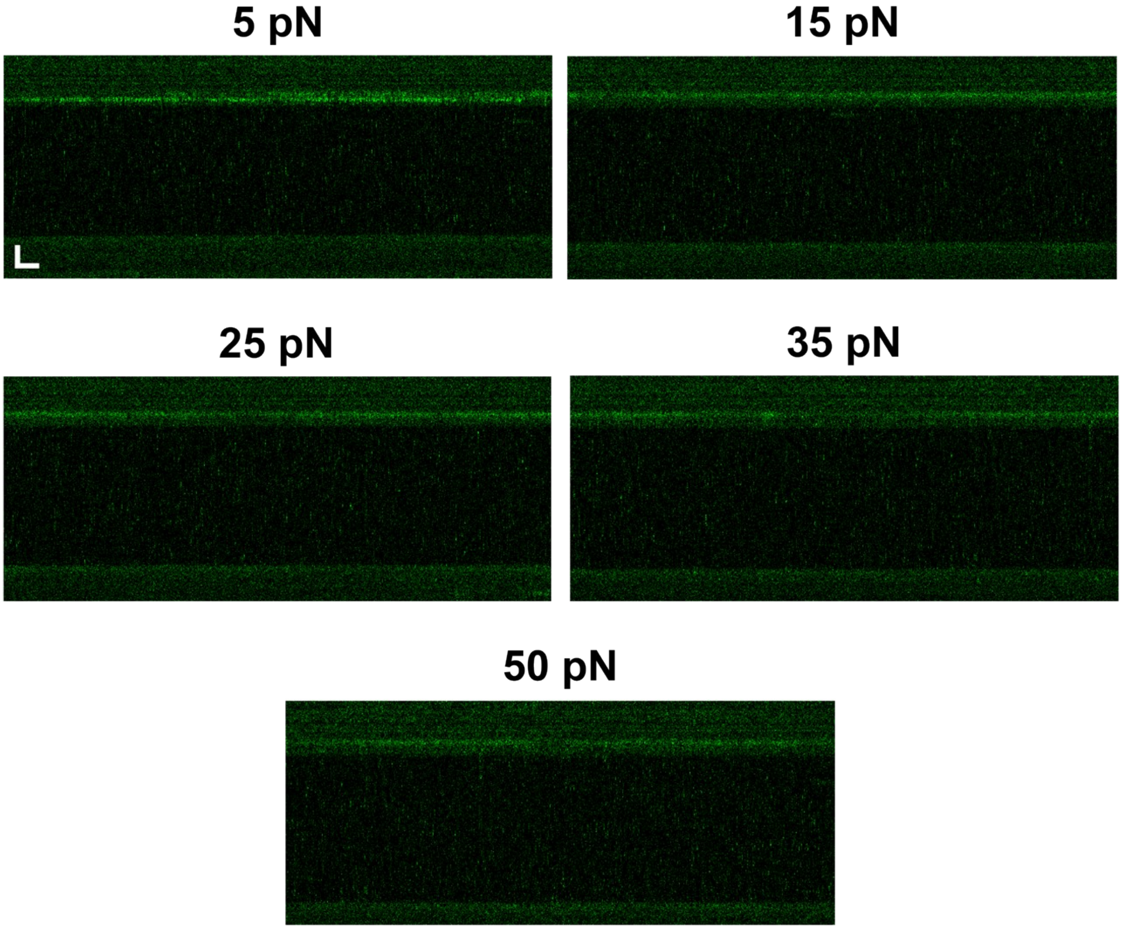

Supplementary Figure 1. Kymographs of force-stretched λ-DNA (5-50pN) in the presence of 1nM crRNA, showing that crRNA alone does not bind the DNA. Scale Bars: 2μm, 2s.

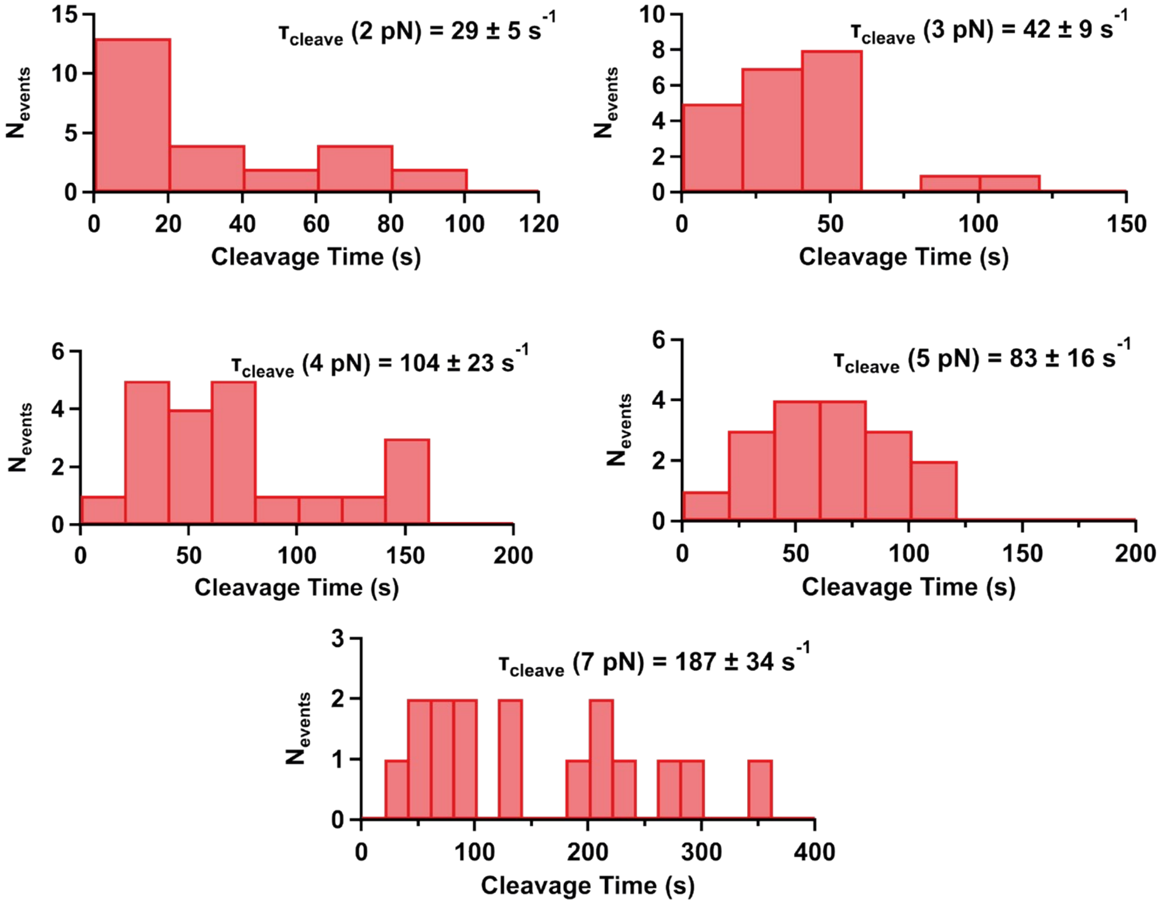

Supplementary Figure 2. Distributions of dwell times between target binding and cutting as a function of force, and average dwell time and standard error of the mean, as indicated. The rate constant k_{cleave} (Figure 2B) was approximated as the inverse of the average dwell time at each force.

Supplementary Movie 1. 2D confocal scan of low force stretched (5pN) λ-DNA incubated with 250 nM Cas12a in a Ca^{2+} buffer (see Methods – Optical tweezers experiments). On target Cas12a is bound stably, and an off-target Cas12a is observed to diffuse one-dimensionally in search for its target.

Supplementary References

1 J. W. Hardin, C. Warnasooriya, Y. Kondo, K. Nagai and D. Rueda, Assembly and dynamics of the U4/U6 di-snRNP by single-molecule FRET., *Nucleic Acids Res.*, 2015, gkv1011.
2 O. Belan, G. Moore, A. Kaczmarczyk, M. D. Newton, R. Anand, S. J. Boulton and D. S. Rueda, Generation of versatile ss-dsDNA hybrid substrates for single-molecule analysis, *STAR Protoc.*, 2021, **2**, 100588.
